# Molecular Characterization of Carotenoid Biosynthetic Genes and Carotenoid Accumulation in *Lycium chinense*

**DOI:** 10.3390/molecules190811250

**Published:** 2014-07-31

**Authors:** Shicheng Zhao, Pham Anh Tuan, Jae Kwang Kim, Woo Tae Park, Yeon Bok Kim, Mariadhas Valan Arasu, Naif Abdullah Al-Dhabi, Jingli Yang, Cheng Hao Li, Sang Un Park

**Affiliations:** 1 Department of Crop Science, College of Agriculture & Life Sciences, Chungnam National University, 79 Daehangno, Yuseong-gu, Daejeon 305-764, Korea; E-Mails: zhaoshicheng@msn.com (S.Z.); tuan_pham_6885@yahoo.com (P.A.T.); harusarinamu@nate.com (W.T.P.); yeonbokkim@hanmail.net (Y.B.K.); 2 Division of Life Sciences, Incheon National University, Yeonsu-gu, Incheon 406-772, Korea; E-Mail: kjkpj@incheon.ac.kr; 3 Department of Botany and Microbiology, Addiriyah Chair for Environmental Studies, College of Science, King Saud University, P. O. Box 2455, Riyadh 11451, Saudi Arabia; E-Mails: mvalanarasu@gmail.com (M.V.A.); naldhabi@ksu.edu.sa (N.A.A.-D.); 4 State Key Laboratory of Tree Genetics and Breeding, Northeast Forestry University, Harbin 150040, China; E-Mail: yifan85831647@sina.com

**Keywords:** carotenoids, gene characterization, lutein, *Lycium chinense*, zeaxanthin

## Abstract

*Lycium chinense* is a shrub that has health benefits and is used as a source of medicines in Asia. In this study, a full-length cDNA clone encoding β-ring carotene hydroxylase (LcCHXB) and partial-length cDNA clones encoding phytoene synthase (LcPSY), phytoene desaturase (LcPDS), ξ-carotene desaturase (LcZDS), lycopene β-cyclase (LcLCYB), lycopene ε-cyclase (LcLCYE), ε-ring carotene hydroxylase (LcCHXE), zeaxanthin epoxidase (LcZEP), carotenoid cleavage dioxygenase (LcCCD1), and 9-*cis* epoxycarotenoid dioxygenase (LcNCED) were identified in *L. chinense*. The transcripts were constitutively expressed at high levels in leaves, flowers and red fruits, where the carotenoids are mostly distributed. In contrast, most of the carotenoid biosynthetic genes were weakly expressed in the roots and stems, which contained only small amounts of carotenoids. The level of *LcLCYE* transcripts was very high in leaves and correlated with the abundance of lutein in this plant tissue. During maturation, the levels of lutein and zeaxanthin in *L. chinense* fruits dramatically increased, concomitant with a rise in the level of β-cryptoxanthin. *LcPSY*, *LcPDS*, *LcZDS*, *LcLCYB*, and *LcCHXE* were highly expressed in red fruits, leading to their substantially higher total carotenoid content compared to that in green fruits. Total carotenoid content was high in both the leaves and red fruits of *L. chinense.* Our findings on the biosynthesis of carotenoids in *L. chinense* provide insights into the molecular mechanisms involved in carotenoid biosynthesis and may facilitate the optimization of carotenoid production in *L. chinense*.

## 1. Introduction

Carotenoids are a family of over 600 plant pigments and are one of the most widespread groups of pigments in Nature [1,2]. In plants, carotenoids play essential roles in photosynthesis, notably light absorption, energy transfer to the reaction center complex, and protection of the photosynthetic apparatus from damage [3,4]. Moreover, carotenoids are intermediates in the biosynthesis of apocarotenoids, which act as plant development signals and antifungal agents and contribute both flavor and aroma to flowers and fruits [5]. Of the over 600 carotenoids found in Nature, approximately 40 are present in a typical human diet. The most widely studied and well-understood nutritional role of carotenoids in humans is their provitamin A activity [6]. A diet high in carotenoids can protect against some cancers, cardiovascular disease, cataracts, and ultraviolet-induced skin damage because these carotenoids act as powerful biological antioxidants [7,8].

**Scheme 1:**
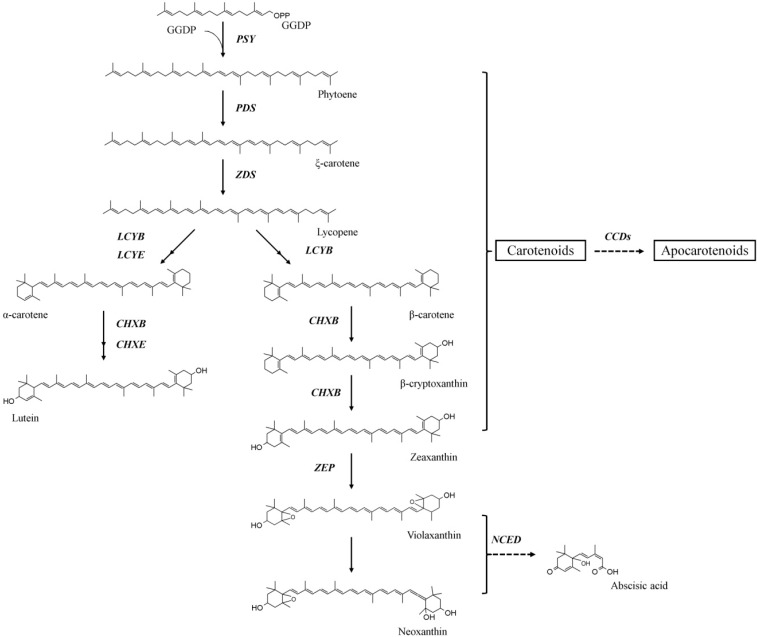
Carotenoid biosynthetic pathway in plants. GGDP, geranylgeranyl diphosphate; PSY, phytoene synthase; PDS, phytoene desaturase; ZDS, ξ-carotene desaturase; LCYB, lycopene β-cyclase; LCYE, lycopene ε-cyclase; CHXB, β-ring carotene hydroxylase; CHXE, ε-ring carotene hydroxylase; ZEP, zeaxanthin epoxidase; CCD, carotenoid cleavage dioxygenase; NCED, 9-cis epoxycarotenoid dioxygenase.

Carotenoids are synthesized in plastids by nuclear-encoded enzymes, and the carotenoid biosynthesis pathway in higher plants is well defined (Scheme 1) [1]. Briefly, phytoene synthase (PSY) catalyzes the condensation of two geranylgeranyl diphosphates to produce phytoene. Phytoene desaturase (PDS) and ξ-carotene desaturase (ZDS) subsequently introduce four symmetric double bonds in phytoene, yielding lycopene via ξ-carotene. Then, lycopene is converted to α-carotene, a process catalyzed by lycopene β-cyclase (LCYB) and lycopene ε-cyclase (LCYE), or to β-carotene, a process catalyzed by LCYB alone. Subsequently, hydroxylation of α-carotene and β-carotene produces either lutein via the activities of β-ring carotene hydroxylase (CHXB) and ε-ring carotene hydroxylase (CHXE) or zeaxanthin via CHXB alone. Zeaxanthin epoxidase (ZEP) metabolizes zeaxanthin to yield violaxanthin. Finally, 9-*cis* epoxycarotenoid dioxygenase (NCED) converts violaxanthin to the plant hormone abscisic acid, ABA [9]. Along the pathway, carotenoids can be cleaved at any of their conjugated double bonds by carotenoid cleavage dioxygenase (CCD) to form a diversity of apocarotenoids [5]. The recent development of next-generation sequencing technologies has provided an excellent research tool for the characterization of a large number of carotenoid biosynthetic genes in non-model plants.

**Figure 1:**
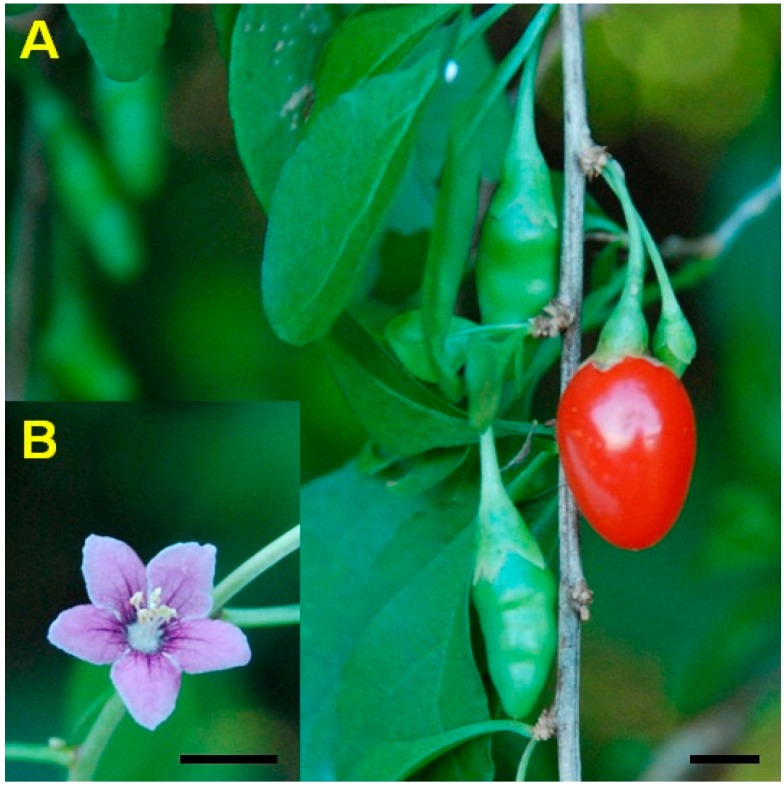
Fruits (**A**) and flower (**B**) of *Lycium chinense*. Bar = 0.5 cm.

*Lycium chinense* (Figure 1)*,* which belongs to the Solanaceae family, has been used in traditional Asian medicine as a medical plant for anti-aging purposes and to reduce the risk of arteriosclerosis and arterial hypertension [10,11]. Numerous pharmacological reports have been published on the biological activities of *Lycium chinense*, which include antioxidant, antiallergic, and anti-inflammatory properties [12,13,14]. A great variety of secondary metabolites have been isolated from *L*. *chinense* plants, including betaine, ascorbic acid, flavonoids, and alkaloids [12,15,16]. Recently, carotenoids from *L*. *chinense* fruits have received attention because of their capacity to reduce myofibroblast-like cell proliferation and induce hepatic fibrosis [17,18]. However, to date, there have been no reports on carotenoid biosynthetic genes or the molecular mechanisms underlying carotenoid biosynthesis in *L*. *chinense*.

In the current study, a full-length cDNA encoding CHXB and partial-length cDNAs encoding PSY, PDS, ZDS, LCYB, LCYE, CHXE, ZEP, CCD1, and NCED were isolated from this plant to investigate the carotenoid biosynthetic pathway in *L. chinense*. Furthermore, the relationship between the transcription levels of carotenoid biosynthetic genes and carotenoid accumulation in the roots, stems, leaves, flowers, green fruits, and red fruits of *L*. *chinense* was examined and has been discussed herein.

## 2. Results and Discussion

### 2.1. Sequence Analyses of Carotenoid Biosynthetic Genes from L. chinense

In another study [19], a 56,526-unigene library were generated from *L. chinense* plants using an Illumina HiSeq 2000 sequencing platform. The carotenoid biosynthetic genes of *Arabidopsis thaliana* obtained from The Arabidopsis Information Resource (TAIR) were used as queries to search for homologous sequences in *L. chinense* transcriptome database. A full-length cDNA clone encoding CHXB and partial-length cDNA clones encoding PSY, PDS, ZDS, LCYB, LCYE, CHXE, ZEP, CCD1, and NCED were identified. They were confirmed for homology with the BLAST program and designated as LcPSY (353 amino acids aa), LcPDS (333 aa), LcZDS (223 aa), LcLCYB (261 aa), LcLCYE (312 aa), LcCHXB (304 aa), LcCHXE (165 aa), LcZEP (178 aa), LcCCD1 (407 aa), and LcNCED (142 aa). The data provided in Table 1 show that the carotenoid biosynthetic genes from *L. chinense* exhibited high identity with other orthologous genes.

**Table 1:** Comparisons of carotenoid biosynthetic genes *L. chinense* with the most orthologous genes.

*L. chinense* (Accession no.)	Length(amino acids)	Orthologous genes(Accession No.)	Identity(%)
*LcPSY*	353	*Nicotiana tabacum PSY2* (JX101474)	93
		*Capsicum annuum PSY1* (EU753855)	94
		*Solanum lycopersicum PSY1* (EF650010)	91
*LcPDS*	333	*Nicotiana benthamiana PDS * (DQ469932)	96
		*Capsicum annuum PDS1*(X68058)	96
		*Solanum lycopersicum PDS* (NM_001247166)	95
*LcZDS*	223	*Capsicum annuum ZDS*(X89897)	95
		*Lycopersicon esculentum ZDS* (DQ412572)	95
		*Solanum lycopersicum ZDS* (NM_001247454)	94
*LcLCYB*	261	*Solanum lycopersicum LCYB* (XM_004249173)	94
		*Capsicum annuum LCYB * (GU085272)	91
		*Solanum lycopersicum LCYB1* (NM_001247297)	90
*LcLCYE*	312	*Nicotiana tabacum LCYE * (HQ993098)	96
		*Solanum lycopersicum LCYE * (EU533951)	96
		*Cucurbita moschata LCYE * (JN559396)	80
*LcCHXB*	304	*Nicotiana tabacum CHXB * (JQ410446)	86
		*Solanum lycopersicum CHXB * (NM_001247419)	89
		*Ipomoea nil CHXB* (B499058)	77
*LcCHXE*	165	*Actinidia chinensis CHXE* (FJ797305)	88
		*Vitis vinifera CHXE* (XM_002264979)	88
		*Cucumis sativus CHXE* (XM_004156280)	85
*LcZEP*	178	*Solanum lycopersicum ZEP* (EU004202)	92
		*Lycopersicon esculentum ZEP* (Z83835)	92
		*Nicotiana plumbaginifolia ZEP* (X95732)	89
*LcCCD1*	407	*Solanum lycopersicum CCD1* (GU120077)	95
		*Petunia x hybrida CCD1* (AY576003)	94
		*Coffea Arabica CCD1* (DQ157170)	85
*LcNCED*	142	*Nicotiana tabacum NCED3* (JX101472)	77
		*Solanum tuberosum NCED1* (AY662342)	76
		*Solanum ochranthum NCED1* (HM156335)	77

### 2.2. Expression Levels of Carotenoid Biosynthetic Genes in Different Organs of L. Chinense

**Figure 2:**
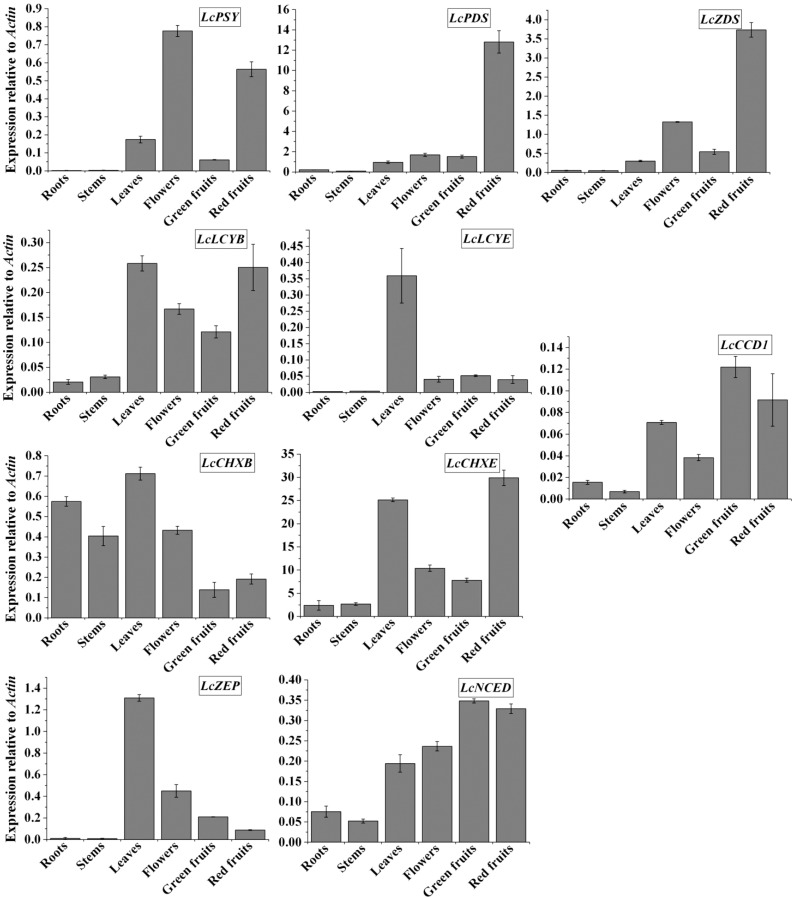
Expression levels of carotenoid biosynthetic genes in different organs of *L. chinense.* The expression levels of carotenoid biosynthetic genes were calculated by the relative quantification method with the *L. chinense* actin housekeeping gene. The height of each bar and the error bars indicate the mean and standard error, respectively, from 3 independent measurements.

The expression of carotenoid biosynthetic genes was analyzed in the roots, stems, leaves, flowers, green fruits, and red fruits of *L. chinense* by real-time RT_PCR (Figure 2). The first committed enzyme in the carotenoid biosynthetic pathway, *LcPSY*, was expressed at the highest level in the flowers, at a relatively high level in the red fruits, at low levels in the leaves and green fruits, and was barely detectable in the roots and stems. Transcription patterns of *LcPDS* and *LcZDS* were essentially similar, with high expression observed in the red fruits and low expression observed in other organs. Expression of *LcLCYB* was high in the leaves and red fruits, moderate in the flowers and green fruits, and poor in the roots and stems. A substantially higher level of *LcLCYE* mRNA expression was detected in the leaves compared to those in the roots, stems, flowers, green fruits, and red fruits. Among the carotenoid biosynthetic genes of *L. chinense*, only *LcCHXB* was highly expressed in the roots and stems. Considerable levels of *LcCHXB* were also detected in the leaves and flowers, whereas only small levels were found in the green and red fruits. *LcCHXE* exhibited high expression levels in the leaves and red fruits, lower levels in the flower and green fruits, and trace levels in the roots and stems. *LcZEP* was expressed strongly only in the leaves and weakly in other organs. Two genes involved in the degradation of carotenoids, *LcCCD1* and *LcNCED*, were expressed at high levels in the green and red fruits, at intermediate levels in the leaves and flowers, and at low levels in the roots and stems. 

### 2.3. Analysis of Carotenoid Content in Different Organs of L. Chinense

The same plant materials used for quantitative real-time RT_PCR were used for the measurement of carotenoid accumulation by using high-performance liquid chromatography with diode-array detection (HPLC_DAD) (Table 2). Only trace amounts of lutein, β-carotene, 9-*cis*-β-carotene, 13-*cis*-β-carotene, and zeaxanthin were detected in the roots. With the exception of lutein (43.49 μg/g dry weight) and β-carotene (18.66 μg/g), no other carotenoids were found in appreciable amounts in the stems. The leaves contained abundant amounts of lutein (1017.66 μg/g) and β-carotene (758.79 μg/g), which are essential for photosynthesis. High amounts of 9-*cis*-β-carotene (64.84 μg/g), 13-*cis*-β-carotene (88.79 μg/g), and zeaxanthin (35.98 μg/g) were also found in the leaves. The majority of carotenoids found in the flowers and green fruits were lutein (120.21 μg/g and 52.89 μg/g, respectively) and β-carotene (77.73 μg/g and 40.14 μg/g, respectively). In contrast to the results for the green fruits, the dominant carotenoids in the red fruits were zeaxanthin, lutein, and β-cryptoxanthin. Specifically, the levels of zeaxanthin, lutein, and β-cryptoxanthin detected in the red fruits were 646.16 μg/g, 168.18 μg/g, and 73.79 μg/g, respectively. In general, carotenoids were mostly distributed in the leaves and red fruits of *L. chinense*, with very high total carotenoid levels of 2,000.73 μg/g and 923.15 μg/g, respectively.

**Table 2:** Carotenoid composition and content in different organs of *L. chinense* (μg/g dry weight). The results have been expressed as mean ± standard error values from 3 independent measurements. N.D. = not detected.

Carotenoids	Roots	Stems	Leaves	Flowers	Green fruits	Red fruits
α-Carotene	N.D.	0.46 ± 0.01	4.76 ± 0.31	0.75 ± 0.04	N.D.	N.D.
Lutein	0.91 ± 0.18	43.49 ± 1.58	1017.66 ± 84.97	120.21 ± 10.28	52.89 ± 10.33	168.17 ± 27.68
β-Carotene	0.49 ± 0.09	18.66 ± 1.70	758.79 ± 19.09	77.73 ± 3.99	40.14 ± 8.14	16.74 ± 4.34
9- *cis* β-Carotene	0.02 ± 0.01	1.64 ± 0.06	64.84 ± 5.78	6.07 ± 0.76	2.47 ± 0.56	11.91 ± 5.88
13- *cis* β-Carotene	0.19 ± 0.01	2.20 ± 0.18	88.79 ± 7.08	8.94 ± 0.58	5.85 ± 0.71	3.42 ± 0.49
β-Cryptoxanthin	N.D.	N.D.	13.47 ± 0.42	0.59 ± 0.04	N.D.	73.49 ± 2.69
Zeaxanthin	0.28 ± 0.13	1.17 ± 0.11	35.98 ± 3.73	8.65 ± 0.85	4.14 ± 0.55	646.16 ± 19.55
Neoxanthin	N.D.	0.31 ± 0.04	16.44 ± 2.77	5.17 ± 0.53	1.54 ± 0.62	3.27 ± 0.58
Total	1.89 ± 0.32	67.94 ± 2.71	2000.73 ± 91.94	228.12 ± 16.93	107.04 ± 19.82	923.15 ± 18.46

### 2.4. Relationship between the Transcription Levels of Carotenoid Biosynthetic Genes and Carotenoid Accumulation in L. chinense

PSY, PDS, and ZDS, which catalyze the first three committed step in carotenoid biosynthesis, were prove to be the major key regulators of carotenoid accumulation in plants [20,21,22]. In *L. chinense*, *LcPSY*, *LcPDS*, and *LcZDS* was highly expressed in red fruits and weakly expressed in the green fruit, roots, and stems. It may lead to substantially higher carotenoid content in the red fruits compared to that in green fruits, roots, and stems. However, the levels of the *LcPSY* transcript were low in the leaves, where abundant amounts of carotenoid were found. Inversely, *LcPSY* was strongly expressed in the flowers, which contained very low levels of carotenoids. This finding suggests the existence of other PSY isoforms in *L. chinense* that control the flux into the carotenoid biosynthetic pathway in leaves and flowers. Two PSY isoforms exist in the tomato, where PSY1 is a fruit- and flower-specific isoform and PSY2 predominates in green tissues [23]. Similarly, the low expression levels of *LcPDS* and *LcZDS* in the leaves suggest the activities of other PDS and ZDS isoforms that contribute to high content of carotenoids in this organ. 

It was observed that the transcription level of *LcLCYE* was very high in the leaves and correlated with the fact that a higher level of lutein was seen in the leaves than in the other organs. We propose that *LcLCYE* regulates the flux of the β,ε-carotenoid branch of carotenoid biosynthesis in the leaves of *L. chinense*. However, low expression levels of *LcLCYE* in the stems, flowers, and green fruits were not associated with the dominant of lutein in these organs. The accumulations of carotenoids in plant tissues are not always correlated tightly with the expression level of carotenoid biosynthetic genes; it might be controlled by additional factors, such as post-transcriptional or metabolic mechanisms. In support of this, the transcription of carotenoid biosynthetic genes was detected in a white carrot cultivar which did not contain carotenoids [24]. 

During the maturation of *L. chinense* fruits, a large increase in the levels of lutein and zeaxanthin, along with the appearance of β-cryptoxanthin were observed. The total carotenoid content increased approximately 9-fold in red fruits compared to that in green fruits. This finding may be attributed to the fact that a higher expression level of *LcPSY*, *LcPDS*, *LcZDS*, *LcLCYB*, and *LcCHXE* was seen in red fruits than in green fruits. When the color of the fruits changes from green to red, the concomitant increase in lutein content is likely due to increased *LcCHXE* expression. However, the 157-fold increase in zeaxanthin and the appeal of β-cryptoxanthin during maturation are not consistent with the slight induction of their biosynthetic gene, *LcCHXB*. As for *LcPSY*, we suggest that there is another isoform of CHXB in *L. chinense*, which directly causes accumulation of β-cryptoxanthin and zeaxanthin in the fruits. In support of this, two types of *CHXB* have been found in the tomato, one of which is specifically expressed in the fruits [25]. It has been reported that ABA plays a crucial role during fruit maturation [26], suggesting that the high expression of *LcNCED* in green and red fruits is responsible for ABA accumulation during fruit maturation in *L. chinense*.

The leaves and fruits of *L. chinense* have been used as foods, tea and/or medicine in the Orient for centuries [10,11]. Here, abundant carotenoid content was found in both the leaves and red fruits of *L. chinense*. The consumption of carotenoids is associated with significant reductions in the risk of lung cancer, prostate cancer, cardiovascular disease, cataracts, and photosensitivity diseases [8,27,28]. Our data therefore provide support for the significant health benefits and medicinal potentials of *L. chinense* plants.

## 3. Experimental Section

### 3.1. Plant Materials

*Lycium chinense*, cultivar Cheongmyeong, was grown in the experimental farm of Chungnam National University (Daejeon, Korea). Six months after sowing, the roots, stems, leaves, flowers, and fruits at 2 different stages, that is, green fruits (immature) and red fruits (mature), were excised. All the samples were immediately frozen in liquid nitrogen and then stored at −80 °C and/or freeze-dried for RNA isolation and/or HPLC_DAD analysis.

### 3.2. RNA Isolation and cDNA Synthesis

The samples were ground into powder in a mortar with liquid nitrogen, and total RNA was isolated separately using a Plant Total RNA Mini Kit (Geneaid, New Taipei City, Taiwan) according to the manufacturer’s instructions. The quality and concentration of total extracted RNA were determined by 1% agarose gel electrophoresis and spectrophotometric analysis, respectively. For first-strand cDNA synthesis, 1 μg of high-quality total RNA was used for reverse transcription (RT) with a ReverTra Ace-R kit (Toyobo Co. Ltd., Osaka, Japan). A 20-fold dilution of 20 μL of the resulting cDNA was used as a template for quantitative real-time RT_PCR.

### 3.3. Sequence Analysis

The deduced amino acid sequences of the carotenoid biosynthesis genes from *L. chinense* were analyzed for homology by using the BLAST program, National Center for Biotechnology Information GenBank database.

### 3.4. Quantitative Real-Time RT_PCR

Based on the sequences of *LcPSY*, *LcPDS*, *LcZDS*, *LcLCYB*, *LcLCYE*, *LcCHXB*, *LcCHXE*, *LcZEP*, *LcCCD1*, and *LcNCED* (accession No. KC810879, KC810880, KC810881, KC810882, KC810883, KC810884, KC810885, KC810886, KC810887, and KC810888), real-time RT-PCR primers were designed using the Primer3 website [29,30] (Table 3). Real-time RT_PCR products were tested for specificity of fragment sizes and melting curves by PCR and real-time PCR, respectively. Subsequently, real-time RT_PCR products were purified and cloning into a T-Blunt vector for sequencing. The expression of these genes was calculated by the relative quantification method with the *L. chinense* actin housekeeping gene (KC810889), which was also isolated in this study (data not shown), as a reference. For quantification of the standard, PCR products amplified from cDNA were purified, and the concentration of the products was measured in order to calculate the number of cDNA copies. Real-time RT_PCR reactions were performed using a 20 μL reaction mix that contained 5 μL of template cDNA, 10 μL of 1X SYBR Green Real-time PCR Master Mix (Toyobo Co. Ltd.), 0.5 μL of each primer (10 μM), and diethylpyrocarbonate-treated water. Thermal cycling conditions were as follows: 95 °C for 5 min and 40 cycles of 95 °C for 15 s, 56 °C for 15 s, and 72 °C for 20 s. PCR products were analyzed with the Bio-Rad CFX Manager 2.0 software [31]. Three replications for each sample were used for the real-time RT_PCR analysis.

### 3.5. Carotenoid Extraction and Analysis

The extraction and measurement of carotenoids by HPLC_DAD were performed as previously described by our group [32,33]. Briefly, carotenoids were released from the *L. chinense* samples (0.02 g) by adding ethanol (3 mL) containing 0.1% ascorbic acid (w/v), vortex mixing for 20 s, and water bath incubation at 85 °C for 5 min. The carotenoid extract was saponified with potassium hydroxide (120 μL, 80% w/v) in the 85 °C water bath for 10 min. After saponification, the samples were immediately placed on ice, and cold deionized water (1.5 mL) was added. *β*-Apo-8′-carotenal (0.2 mL, 25 g/mL) was added as an internal standard. Carotenoids were extracted twice with hexane (1.5 mL) by centrifugation at 1,200× *g* in order to separate the layers. Aliquots of the extracts were dried under a stream of nitrogen and redissolved in 50:50 (v/v) dichloromethane/methanol before analysis by HPLC_DAD. The carotenoids were separated on a C30 YMC column (250 × 4.6 mm, 3 μm; Waters Corporation, Milford, MA, USA) by an Agilent 1100 HPLC system (Agilent Technologies France, Massy, France) that was equipped with a photodiode array detector. Chromatograms were generated at 450 nm. Solvent A consisted of methanol/water (92:8 v/v) with 10 mM ammonium acetate. Solvent B consisted of 100% methyl *tert*-butyl ether. Gradient elution was performed at 1 mL/min under the following conditions: 0 min, 90% A/10% B; 20 min, 83% A/17% B; 29 min, 75% A/25% B; 35 min, 30% A/70% B; 40 min, 30% A/70% B; 42 min, 25% A/75% B; 45 min, 90% A/10% B; and 55 min, 90% A/10% B. Carotenoid standards were purchased from CaroteNature GmbH (Lupsingen, Switzerland). For quantification purposes, calibration curves were drawn by plotting 4 different concentrations of carotenoid standards according to the peak area ratios with *β*-apo-8′-carotenal.

**Table 3:** Primers used for real-time RT_PCR.

Gene	Forward (5′ to 3′)	Reverse (5′ to 3′)
*LcPSY*	AGCAAATCCAGAGAGCAAGAAAGTT	GTAGTCATTGGCTTCAATTTCATCG
*LcPDS*	CCCCAATAGAGGGGTTTTATTTAGC	CTGTAAAATAGCTTGCGCACAGAGT
*LcZDS*	CCTTACATGCCTCTACCAAATGATG	ATACAAGGATTGCCCAATTTTCACT
*LcLCYB*	GGATTGGCGAGATTCTCATCTTAAT	CTTAAACGAGCCACCATTCTTTCTT
*LcLCYE*	ACAGCTGGATATTGAGGGAATAAGG	CTCATGTCATTTGGTGCAATGATAA
*LcCHXB*	ACATGTTCGTTCACGATGGTTTAGT	CTCTTCAAGTCCTCCTACGTCTTCC
*LcCHXE*	TCTTTGGAAAAAGCACATGAAGAAG	TCTTATTAGGACAGGTGGATGTGGA
*LcZEP*	ATGATGATGCTTTAGAGCGTGCTAC	AGACCCAATAGTGCAAGAGATACCC
*LcCCD1*	GATCTTAAAGGGCTGTTTGGTCTGT	CGTATTAGCTGTGCCATTTCCATAG
*LcNCED*	TCCACCTATTCTCCATTTCCCTAAA	GGAGGATTTATTTTCTTGCTTTGGA
*LcActin*	ACCACTTGTTTGTGACAATGGAACT	TCAATTGGGTATTTCAAGGTCAAGA

## 4. Conclusions

The molecular characterization of carotenoid biosynthetic genes and analyses of carotenoid accumulation provides insights into the molecular basis underlying carotenoid biosynthesis in *L. chinense*. Our findings regarding the abundant carotenoid content in the leaves and fruits provide evidence to support that carotenoids contribute to the remarkable biological activities of *L. chinense*.
